# Toxic Epidermal Necrolysis Secondary to Iodine Versus Methimazole in a Pediatric Patient With Complex Autoimmune Disease

**DOI:** 10.7759/cureus.57618

**Published:** 2024-04-04

**Authors:** Katlyn M Smaha, John D Prosser, Jacqueline T Chan

**Affiliations:** 1 Pediatric Endocrinology, Medical College of Georgia at Augusta University, Augusta, USA; 2 Pediatric Otolaryngology, Head and Neck Surgery, Medical College of Georgia at Augusta University, Augusta, USA; 3 Pediatric Endocrinology, University of Utah Health, Salt Lake City, USA

**Keywords:** graves’ disease, autoimmune disease, methimazole, potassium iodide, adverse drug reaction

## Abstract

We report a case of a 17-year-old girl who developed toxic epidermal necrolysis (TEN) secondary to preoperative iodine administration before thyroidectomy for Graves’ disease. Past medical history was significant for COVID-19 and multisystem inflammatory syndrome in Children (MISC-C), with subsequent diagnoses of type 1 diabetes mellitus (T1DM), Addison disease, and Graves' disease. Her Graves disease was initially managed with methimazole. While there are reported cases of Stevens-Johnson syndrome (SJS) and TEN due to methimazole, the patient had discontinued methimazole over one month prior. Therefore, she likely represents the first case of TEN reported secondary to potassium iodide solution in a pediatric patient. Given the rarity of TEN in pediatric patients, our case highlights the challenges in managing complex autoimmune conditions and underscores the importance of careful medication choices in such cases.

## Introduction

Graves’ disease is the leading cause of hyperthyroidism in children and adolescents [1]. There are three treatment options: antithyroid drugs (ATDs), radioactive iodine ablation, or thyroidectomy [2,3]. ATD therapy, particularly methimazole, is the preferred initial treatment due to its safer side effect profile [2]. When ATDs fail to resolve symptoms or lead to intolerable adverse effects, thyroidectomy or radioactive iodine ablation is indicated [3]. Before undergoing thyroidectomy, patients typically receive potassium iodide solutions, like Lugol's solution, to reduce bleeding complications and the risk of thyroid storm [2,4].

Stevens-Johnson syndrome (SJS) and toxic epidermal necrolysis (TEN) are severe skin and mucous membrane reactions often associated with medication use [5]. While there have been no prior reports of SJS/TEN linked to preoperative iodine solutions, there have been four documented cases of these conditions resulting from methimazole therapy in pediatric patients [6,7]. Notably, in those instances, the patients were actively taking methimazole when SJS/TEN developed [6,7].

We present a case of a 17-year-old girl with a complex history of polyglandular autoimmune syndrome (PAS) type 2 who developed TEN following preoperative iodine administration in preparation for thyroidectomy to treat Graves' disease. The patient had discontinued methimazole over one month prior to the onset of symptoms, making this case the probable first instance of TEN linked to potassium iodide solution in a pediatric patient.

This article was previously presented as a meeting abstract at the 2023 Pediatric Endocrine Society Annual Meeting on May 7, 2023.

## Case presentation

A 17-year-old girl with a past medical history of COVID-19, multisystem inflammatory syndrome in children (MIS-C), Graves’ disease, type 1 diabetes mellitus (T1DM), and Addison disease presented to the emergency department for evaluation of a diffuse, pruritic rash developing five days after starting iodine solution per thyroidectomy protocol for Graves' disease. She reported fever, malaise, and burning and tingling of her skin one day prior to the onset of her rash. 

Two months prior to presentation, she was diagnosed with T1DM, Addison disease, and Graves' disease fulfilling the clinical diagnosis of PAS type 2. Her T1DM was well controlled with insulin. She was taking prednisone and fludrocortisone for her Addison's disease.

The management of her Graves' disease started with propranolol and methimazole but was complicated by a pruritic, erythematous, macular rash that developed two days after starting methimazole. Despite diphenhydramine premedication and dose reduction of methimazole, the rash persisted. Additionally, she developed a diffuse goiter two weeks after her Graves diagnosis, leading to a shared decision to plan for a definitive thyroidectomy. Methimazole was stopped, which led to the complete resolution of her rash and pruritus. Her hyperthyroidism was controlled with prednisone and propranolol while awaiting surgery, which was delayed by one month due to scheduling backup. One week before the scheduled surgery, she began iodine solution. Of note, the patient's father had a history of allergy to iodinated contrast media that was not known prior to administration of iodine solution.

**Figure 1:**
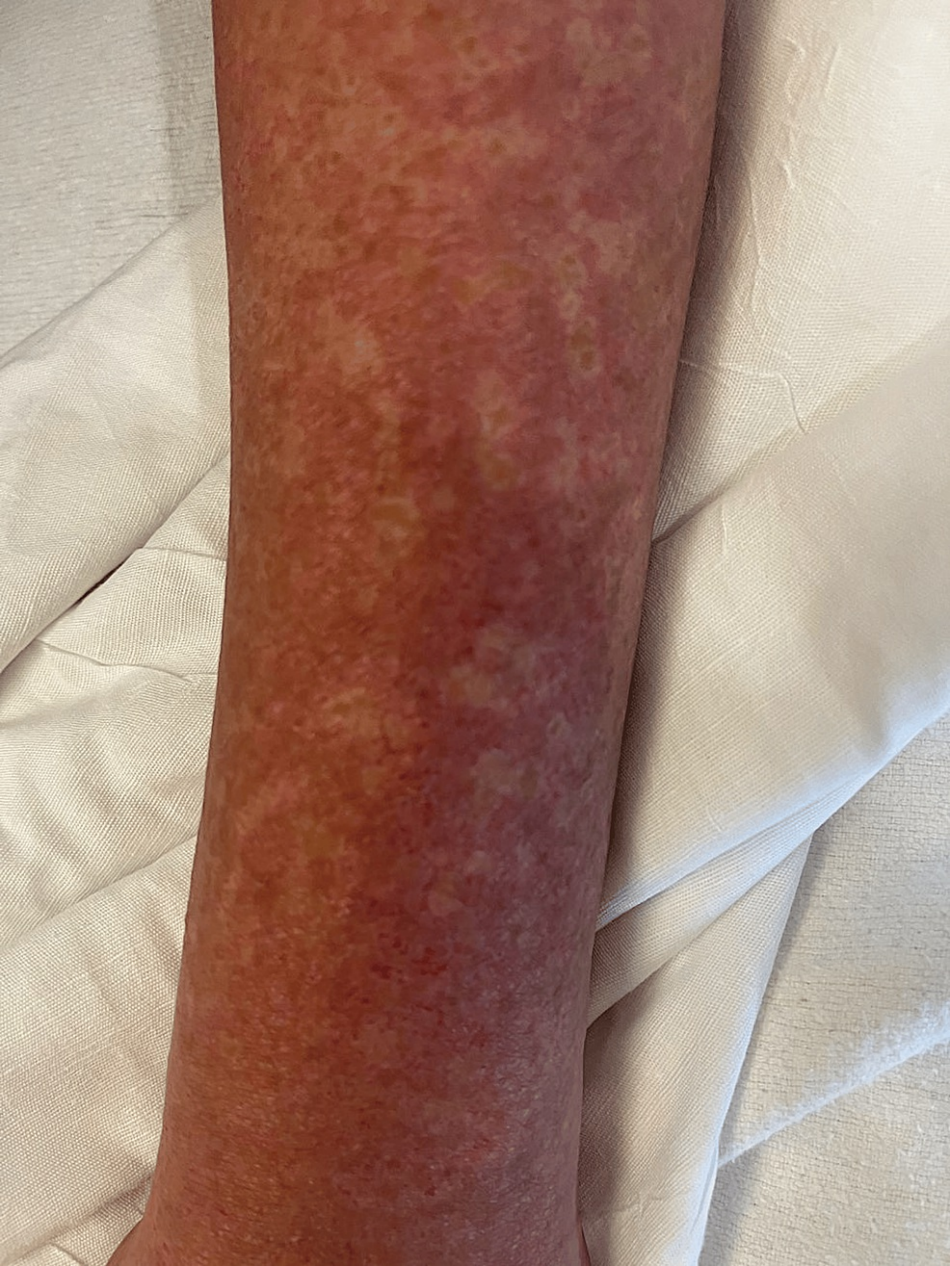
Left forearm with painful, morbilliform rash on arrival to the emergency department on day 5 after starting potassium iodide solution.

On her fifth day of iodine solution, she presented to the emergency department with a new onset, diffuse, pruritic morbilliform eruption (Figure 1). On presentation, she was tachycardic and febrile at 102 °F. Prednisone was switched to intravenous hydrocortisone at stress dose, and iodine was discontinued. She was admitted for concerns of thyroid storm, although her TSH and free T4 levels were improved from a month prior. An urgent thyroidectomy was performed, which the patient tolerated well. 

**Figure 2:**
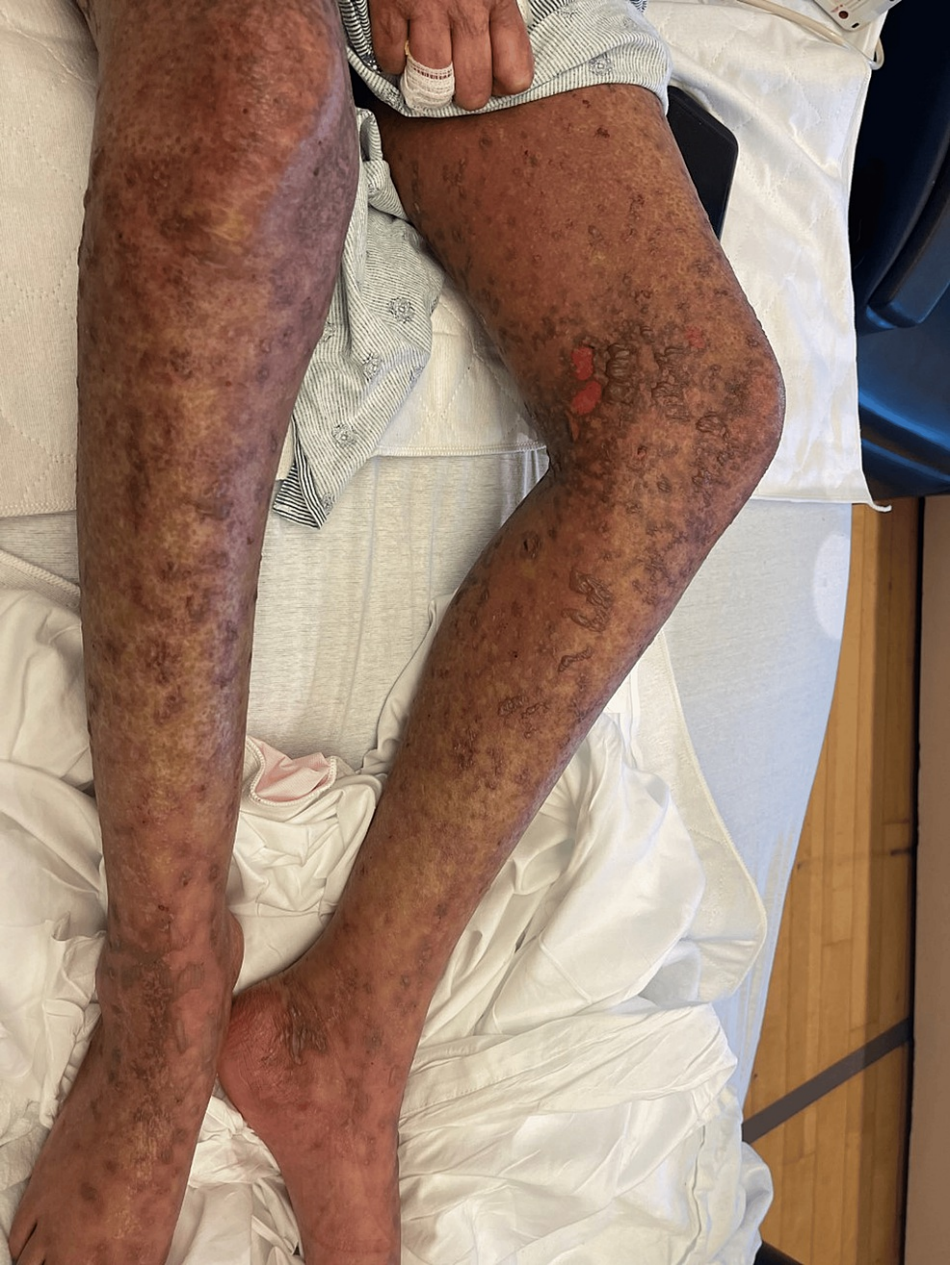
Bilateral lower extremities with diffuse, dusky red to purpuric, coalescing macules with positive Nikolsky on day 7 after starting potassium iodide solution.

**Figure 3:**
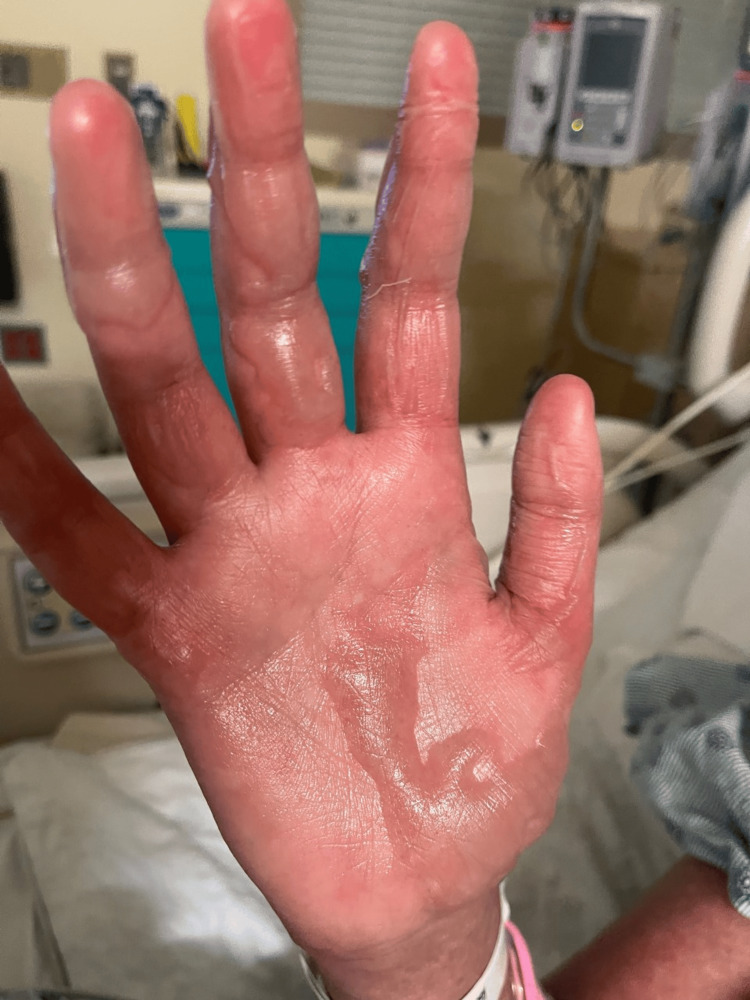
Palm of right hand with diffuse, painful blistering on day 7 after starting potassium iodide solution.

Twenty-four hours post-operatively, her rash progressed with new skin sloughing, leading to a dermatology consultation. On exam, diffuse dusky red to purpuric macules were present on her face, back, chest, abdomen, and bilateral legs and arms. Positive Nikolsky sign was present on bilateral upper and lower extremities (Figure 2). There was a small erythematous ulcer at the left oral commissure but no lesions on the rest of her mouth, tongue, vulvar, or perianal areas. There was painful, diffuse blistering of bilateral palms and soles (Figure 3). Eye involvement was spared.

Given diffuse, blistering rash with positive Nikolsky sign, mucosal involvement, and fever, the patient was diagnosed with SJS likely secondary to recent administration of iodine solution. Due to the known benefit of intravenous immunoglobulin (IVIG) when given early and the patient’s history of tolerating IVIG in the past for MIS-C, a biopsy was not performed in favor of starting high-dose IVIG (2 g/kg totally) quickly to minimize the risk of progression to TEN.

Three days after admission, she developed painful ulcers with white to gray shallow bases on her tongue as well as painful vaginal mucosal erosions with scant bleeding. Ophthalmology was consulted due to erythematous papules on upper eyelids, but it was determined that her eyes were spared. Her symptoms progressed with blistering and sloughing of the skin affecting 85% of her body approximately 10 days after starting the iodine solution, leading to a diagnosis of TEN. She was transferred to a burn unit facility for surgical debridement and continued management with high-dose IVIG therapy.

The patient was hospitalized for 38 days. She was discharged stable but still requires a wheelchair due to foot pain. She followed up with endocrine and dermatology following discharge. Three months following discharge, her skin was healed with post-inflammatory hyperpigmentation and scarring present on most of her body. She was ambulating well at this follow-up visit.

## Discussion

SJS and TEN are serious and life-threatening mucocutaneous conditions usually due to adverse drug reactions, with rare incidence in children [8]. A prodrome of “influenza-like” symptoms is usually followed by the development of painful blisters and widespread skin detachment that exhibit the Nikolsky sign [5]. As seen in our patient, tense vesicles or bullae may be observed, typically on the palms and soles as their thicker epidermal layer more readily resists pressure [9]. SJS/TEN are defined on a spectrum by the percentage of skin detachment, with SJS affecting <10%, TEN >30%, and SJS/TEN overlap involving 10%-30% of the patient's skin [5]. Skin biopsy usually shows full-thickness epidermal necrosis with lymphocytic dermal inflammation, although it is not required for diagnosis [8].

Although the pathogenesis is not fully understood, SJS and TEN are classified as type IV hypersensitivities, with CD8+ cytotoxic T cells acting as the major mediator of keratinocyte death [10]. TEN usually occurs between seven days and eight weeks after drug ingestion, with the average onset occurring between six days and two weeks [11]. Our patient’s symptoms continued to progress after discontinuation of the iodine solution, with a final diagnosis of TEN made approximately 10 days following iodine ingestion.

Sassolas et al. developed an algorithm of drug causality for epidermal necrolysis (ALDEN), which scores drugs from -1 to 10 based on six parameters (Table 1) [12]. Given that methimazole was stopped before the index day by >5 times its elimination half-life, it is unlikely that this medication was still present in our patient’s body when symptoms first began. Using this algorithm, potassium iodide solution is more likely the culprit medication, though methimazole cannot be completely ruled out.

**Table 1:** Algorithm of drug causality for epidermal necrolysis (ALDEN) scoring system. EuroSCAR, European severe cutaneous adverse reaction; SJS, Stevens-Johnson syndrome; TEN, toxic epidermal necrolysis. * Reprinted from Schwartz RA, McDonough PH, Lee BW, Toxic epidermal necrolysis: Part I. Introduction, history, classification, clinical features, systemic manifestations, etiology, and immunopathogenesis, J Am Acad Dermatol, 69(2), 173-186, 2013, with permission from Elsevier [OR APPLICABLE SOCIETY COPYRIGHT OWNER].

Criteria*	Score
Period between the initial drug intake and onset of reaction (index day)	
5-28 days	+3
29-56 days	+2
1-4 days	+1
>56 days	-1
Drug started on index day	-3
With previous history of adverse reaction from same drug, 1-4 days	+3
With previous history of adverse reaction from same drug, 5-56 days	+1
Presence of drug in the body on index day	
Stopped on the index day or within 5 times the elimination half-life before the index day	0
Stopped at a time point before the index day by >5 times the elimination half-life with presence of liver or kidney dysfunction	-1
Stopped at a time point before the index day by >5 times the elimination half-life	-3
Previous history of adverse reaction	
SJS/TEN from same drug	+4
SJS/TEN from similar drug	+2
Other reaction from similar drug	+1
No history of exposure to the drug	0
Previous use without any reaction	-2
Continued drug use beyond index day	
Stopped (or unknown)	0
Continued without harm	-2
Drug notoriety derived from previous results of the EuroSCAR study [13]	
“High risk”	+3
“Lower risk”	+2
“Under surveillance,” meaning several previous reports but unclear epidemiology results	+1
All other drugs, including newly released drugs	0
“No evidence of association” from previous epidemiology study	-1
Other possible etiologic alternatives	
Infectious agent	-1
If the patient is taking multiple drugs and at least 1 drug has a score >3, subtract 1 point from each of the other drugs	-1
The score is categorized as very probable (≥6), probable (4-5), possible (2-3), unlikely (0-1), and very unlikely (<0) [12].	

Drugs with a high risk of causing SJS/TEN include allopurinol, carbamazepine, fluoroquinolones, lamotrigine, minocycline, nevirapine, nonsteroidal anti-inflammatory drugs, phenobarbital, phenytoin, sulfasalazine, and trimethoprim-sulfamethoxazole [13]. There are reported cases of SJS/TEN secondary to iodinated contrast agents [14]. However, preoperative iodine preparations have a low frequency of adverse effects, with no reported cases of SJS/TEN [15]. In high doses, they may cause side effects such as fever, weakness, swelling, mouth sores, skin rash, nausea, vomiting, stomach pains, or numbness or tingling in the hands or feet [15]. In a study by Calissendorff et al., 15% of patients treated with iodine solution for uncontrolled hyperthyroidism experienced mild adverse effects, primarily in the form of a rash [16].

Other risk factors for SJS/TEN include autoimmune disease, active malignancy, genetic predisposition, and immune dysregulation, such as HIV infection [17,18]. Autoimmune diseases linked to SJS/TEN include systemic lupus erythematosus and autoimmune polyglandular syndrome type I [17,19]. In such diseases, SJS/TEN can occur even if the patient is currently on corticosteroid treatment [19]. Our patient may have been at increased risk of adverse effects from iodine solution due to her autoimmune disease and a significant family history of allergy to iodinated contrast media.

Treatment of SJS/TEN in pediatric patients aligns with adult strategies, including discontinuation of suspected causative agents, supportive care, and consideration of systemic corticosteroids and IVIG [8,20]. To reduce complications of the loss of barrier function, wound debridement, removal of necrotic skin, and use of topical antiseptics/antibiotics may be needed [8,20].

## Conclusions

Our patient’s case is unique and raises the question of whether her SJS/TEN resulted from methimazole or iodine solution. She had been discontinued from methimazole for over one month when symptoms of SJS/TEN developed. Due to these conditions and family history of iodine allergy, our patient is more likely the first case of SJS/TEN seen due to preoperative iodine solution. Her history of autoimmune disease may have put her at greater risk for adverse drug reaction and highlights the challenges in managing autoimmune conditions in pediatric patients.
